# Investigation of Antiparasitic Activity of 10 European Tree Bark Extracts on Toxoplasma gondii and Bioguided Identification of Triterpenes in *Alnus glutinosa* Barks

**DOI:** 10.1128/AAC.01098-21

**Published:** 2022-01-18

**Authors:** Pierre Darme, Jérémy Spalenka, Jane Hubert, Sandie Escotte-Binet, Laurent Debelle, Isabelle Villena, Charlotte Sayagh, Nicolas Borie, Agathe Martinez, Benjamin Bertaux, Laurence Voutquenne-Nazabadioko, Jean-Hugues Renault, Dominique Aubert

**Affiliations:** a Université de Reims Champagne-Ardenne, ESCAPE EA 7510, Centre Hospitalier de Reims et Université de Reims Champagne-Ardenne, Reims, France; b Université de Reims Champagne-Ardenne, CNRS, ICMR 7312, Reims, France; c NatExplore SAS, Prouilly, France; d Université de Reims Champagne Ardenne, CNRS, MEDyC UMR 7369, Reims, France; e Centre National de Référence de la Toxoplasmose, Centre de Ressources Biologiques Toxoplasma, Centre Hospitalier de Reims et Université de Reims Champagne-Ardenne, Reims, France

**Keywords:** *Toxoplasma gondii*, *Alnus glutinosa*, Betulaceae, triterpene, apicomplexa, bark, antiparasitic agents

## Abstract

Toxoplasmosis is a worldwide parasitosis that affects one-third of the population. People at risk, such as immunocompromised patients (AIDS, chemotherapy treatment) or fetuses (maternal-fetal transmission) can develop severe forms of the disease. The antiparasitic activity of extracts of different polarities (*n*-heptane, MeOH, MeOH/H_2_O) of 10 tree species endemic to temperate regions was investigated against Toxoplasma gondii infection *in vitro*. Our results showed that the *n*-heptane extract of the black alder (*Alnus glutinosa*) exhibited a significant antiparasitic activity without any cytotoxicity at the tested concentrations, with an IC_50_ of up to 25.08 μg/mL and a selectivity index higher than 3.99. The chemical profiling of this extract revealed triterpenes as major constituents. The ability of commercially available triterpene (betulin, betulinic acid, and betulone) to inhibit the growth of T. gondii was evaluated and showed growth inhibition rates of 44%, 49%, and 99% at 10 μM, respectively.

## INTRODUCTION

Toxoplasmosis is a worldwide disease caused by the protozoan parasite Toxoplasma gondii, which belongs to the Apicomplexa phylum. Toxoplasmosis is one of the most common parasitic infections, generally benign in immunocompetent individuals. However, severe effects can be observed in the case of mother-to-child transmission of the parasite during pregnancy of primo-infected women (congenital toxoplasmosis) (1) or in the case of reactivation of a previous infection triggering cerebral or global toxoplasmosis in immunocompromised patients (2).

The therapeutic armamentarium against Toxoplasma gondii is relatively poor and old, and presents side effects, especially the pyrimethamine and sulfadiazine combination. Moreover, parasites can develop a resistance against drugs, as demonstrated *in vitro* for T. gondii (3–5). Some T. gondii strains can also naturally present different susceptibility toward drugs (6, 7). Due to the lack of specificity (available drugs are limited and none of them are specific to toxoplasmosis) and limited efficacy of the current treatments, new active compounds are needed to treat toxoplasmosis.

The use of plants for therapeutic purposes has always existed in human and animal behaviors. Newman and Cragg reported that between 1981 and 2019, 20 new antiparasitic drugs were discovered, of which two are natural products, seven are compounds derived from natural products, and three are synthetic drugs with a natural pharmacophore (8). Traditional medicine is also still very present in some countries, especially to treat parasitic diseases such as trypanosomiases (9) or malaria (10). Many treatments currently used against diverse pathologies have a natural origin, such as artemisinin from *Artemisia annua* (11) and quinine from *Cinchona officinalis* (12) against malaria, or paclitaxel from *Taxus baccata* against cancer (13). For 20 years, one can note a significant increase in the number of publications relating to the search for new compounds to fight against T. gondii. In this field of research, works on new anti-*toxoplasma* molecules derived from terrestrial plants, marine organisms, microorganisms, and even animals have intensified in the last 10 years, some of them being based on ethnopharmacological considerations (14, 15). To our knowledge, no natural product currently exists that was patented as an anti-*toxoplasma* active substance.

In the present study, 10 tree species with high occurrence in European forests were investigated for their anti-T. gondii activities: Fagus sylvatica L. (common beech, Fagaceae), Quercus robur L. (common oak, Fagaceae), *Alnus glutinosa* (L.) Gaertn (black alder, Betulaceae), Prunus avium (L.) L. (wild cherry, Rosaceae), *Acer pseudoplatanus* L., (sycamore maple, Sapindaceae), *Fraxinus excelsior* L. (common ash, Oleaceae), *Populus tremula* L. (common aspen, Salicaceae), *Populus x canescens* Aiton (robusta poplar, Salicaceae), *Larix decidua* Mill. (European larch, Pinaceae), and *Picea abies* (L.) H.Karst (Norway spruce, Pinaceae).

Since barks constitute an interface between the tree heart and its environment, metabolites extracted from barks can lead to the discovery of protective agents against diverse pathogens or aggressions (16). Therefore, several extracts were produced from the barks of the 10 European tree species and tested *in vitro* to evaluate their activity against T. gondii growth inhibition and cytotoxicity on non-infected Vero cells.

Then a ^13^C NMR-based (Nuclear Magnetic Resonance) dereplication workflow combined with a bioactivity-guided fractionation process was applied on the most active extract to identify the compounds being involved in the biological effect against T. gondii. In addition, the screening of four commercially available identified compounds was performed at 1 and 10 μM.

## RESULTS

### Solid-liquid extraction of European tree barks.

The 10 different barks were macerated at room temperature successively with *n*-heptane, MeOH, and MeOH-water 1:1 (vol/vol) (17) yielding 30 solid-liquid crude extracts. Yields are presented in Table S1.

### Biological screening of the barks extracts on T. gondii.

**FIG 1:**
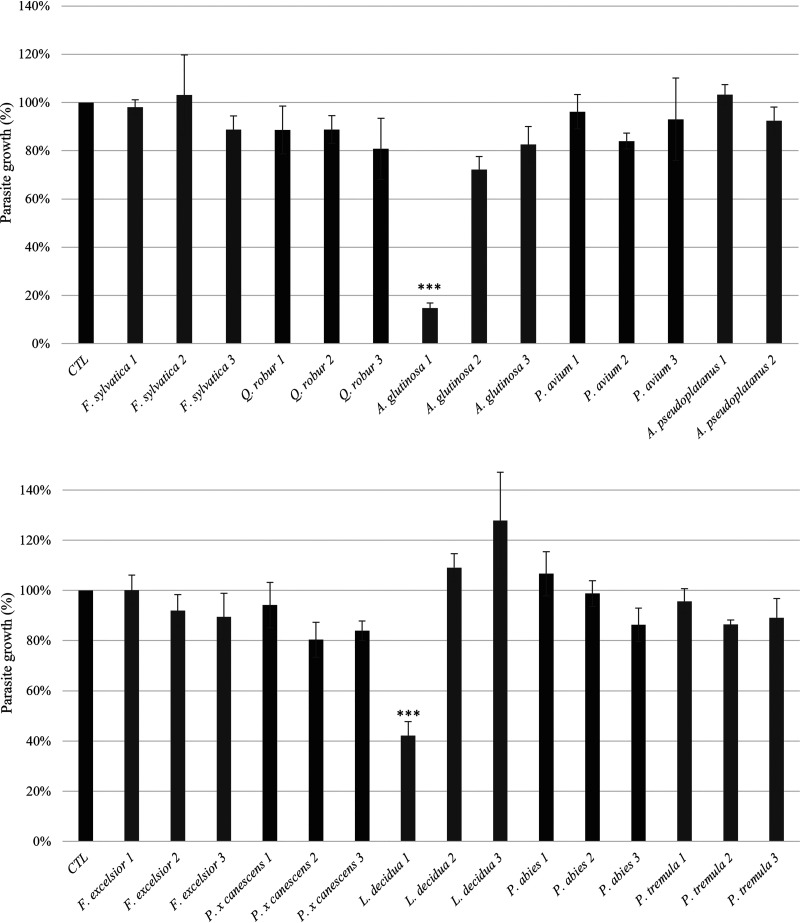
*In vitro* screening of bark extracts on T. gondii tachyzoites (RH strain) growth after 72 h of incubation. Each graph shows the tachyzoites growth compared to positive control and determined by enzyme immunoassay with infected monolayers (*y* axis) versus bark extract (*x* axis). Bark extracts were tested at 100 μg/mL. Numbers 1, 2, and 3 respectively stand for *n*-heptane, MeOH and MeOH/H_2_O extracts.

*n*-Heptane, MeOH, and MeOH/water 1:1 (vol/vol) bark extracts were all tested on T. gondii (Fig. 1) at 100 μg/mL, except the *A. pseudoplatanus* methanol/water 1:1 (vol/vol) extract that was not soluble in DMSO.

Fig. 1 shows that the *n*-heptane extract of *A. glutinosa* and *L. decidua* showed the highest activity against T. gondii with 85% and 58% growth inhibition, respectively. These observations were confirmed microscopically. On the contrary, MeOH and MeOH/H_2_O extracts showed no significant activity against T. gondii tachyzoites. The *n*-heptane extract of *A. glutinosa* that presented the most promising activity was further fractionated by CPC and chemically profiled based on the ^13^C NMR data (18) to identify the compounds responsible for the antiparasitic activity.

### Fractionation of the *n*-heptane extract of *A. glutinosa* by CPC.

**FIG 2:**
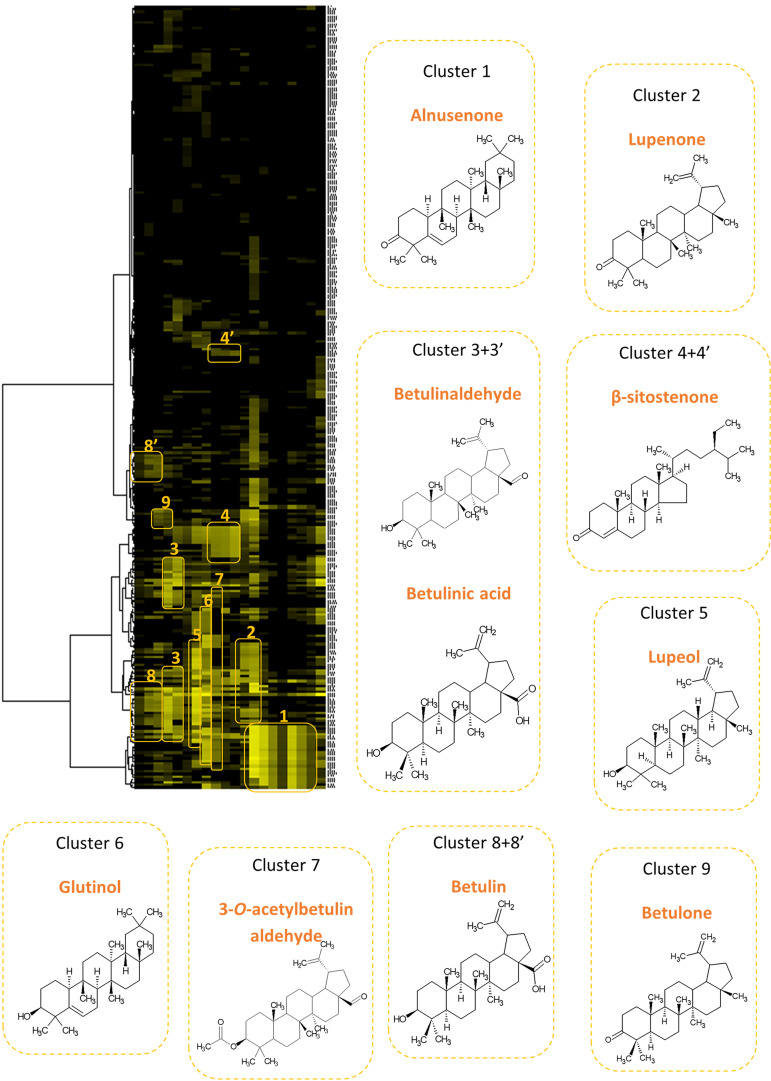
Heatmap of peak intensities of ^13^C NMR (rows) and fractions (columns) from *Alnus glutinosa n*-heptane extract. This representation allows the visualization of the carbon skeleton of the major compounds. The 9 clusters represent the 10 major compounds identified by the CARAMEL dereplication procedure. The molecules were designed with ChemDraw 18.0.

The *n*-heptane extract of *A. glutinosa* was fractionated by CPC, yielding 19 chemically simplified fractions, 16 corresponding to the elution step and the last 4 being obtained after a stationary phase extrusion step (19). The chemical profiling of the CPC fractions was achieved by a dereplication process based on unsupervised clustering of recorded ^13^C NMR data (20, 21). The resulting heat map after hierarchical clustering analysis on the ^13^C chemical shifts (i.e., on the lines of the table) highlights nine clusters (Fig. 2). The chemical shifts corresponding to each cluster were one by one submitted to an in-house database. This database contains more than 3,000 compounds to date and associates natural product structures to the predicted ^1^H and ^13^C NMR chemical shifts calculated by the ACD/Labs predictor. A literature survey was carried out on the plant *A. glutinosa*, resulting in 68 metabolites stored in the database. As a result, 10 triterpenes—major constituents of the *n-*heptane bark extract—were identified and confirmed by interpretation of 1D and 2D NMR data. Fractions 1–5 corresponded to mixtures of betulin (cluster 8 and 8′), betulone (cluster 9), and betulinaldehyde and betulinic acid (cluster 3 and 3′) at different ratios (Fig. 2). The main compounds in fraction 7 and fraction 8 are lupeol (cluster 5) and glutinol (cluster 6), respectively. Fraction 9 mainly contains β-sitostenone (cluster 4 and 4′) and 3-*O*-acetylbetulin-aldehyde (cluster 7), whereas β-sitostenone is the major compound in fractions 10–11. Fraction 12 is mainly composed of lupenone (cluster 2), the latter being in a mixture with alnusenone (cluster 1) in fraction 13. Finally, alnusenone is the major compound in fractions 14–19. The composition of fraction 20 corresponded almost to the composition of the initial extract, a phenomenon frequently encountered and probably due to a dead volume in the line of fluid discharged during the extrusion step. Fraction 20 was excluded from this study. Fraction compositions are summarized in Table 1.

**TABLE 1:** Activity of the selected fractions (> 50% of parasite growth inhibition at 25 μg/mL) obtained from the *n*-heptane bark extract of *A. glutinosa* on Vero cells (CC_50_), T. gondii (IC_50_), and their respective selectivity indexes (SI)^*a*^

*A. glutinosa* active fractions (from *n*-heptane extract)	Main compounds	CC_50_ Vero cells (μg/mL)	IC_50_ T. gondii (μg/mL)	SI T. gondii
F1	Betulin, betulinaldehyde, betulone and betulinic acid	62.03	7.24 ± 1.45	8.57
F3		58.23	3.31 ± 0.12	17.62
F4		42.93	2.64 ± 0.61	16.26
F5		44.76	3.85 ± 0.36	11.64
F9	β-sitostenone and 3-*O*-acetylbetulin-aldehyde	54.41	6.25 ± 0.83	8.71
F10	β-sitostenone	102.01	7.11 ± 1.06	14.35
F11		148.48	6.21 ± 1.77	23.93
F12	Lupenone	58.47	2.95 ± 1.42	19.85
F13	Lupenone and alnusenone	>100	13.51 ± 4.34	>7.40
F14	Alnusenone	>100	21.50 ± 7.08	>4.65
Extract	-	>100	25.08 ± 4.63	>3.99

a Values are expressed as mean ± SD. Underlined values are theoretical based on the trend line since CC_50_ was not reached. The main compounds contained in the *n*-heptane bark extract of *A. glutinosa* after its fractionation by CPC were annotated based on the ^13^C NMR dereplication workflow.

### Cytotoxicity assay.

**FIG 3:**
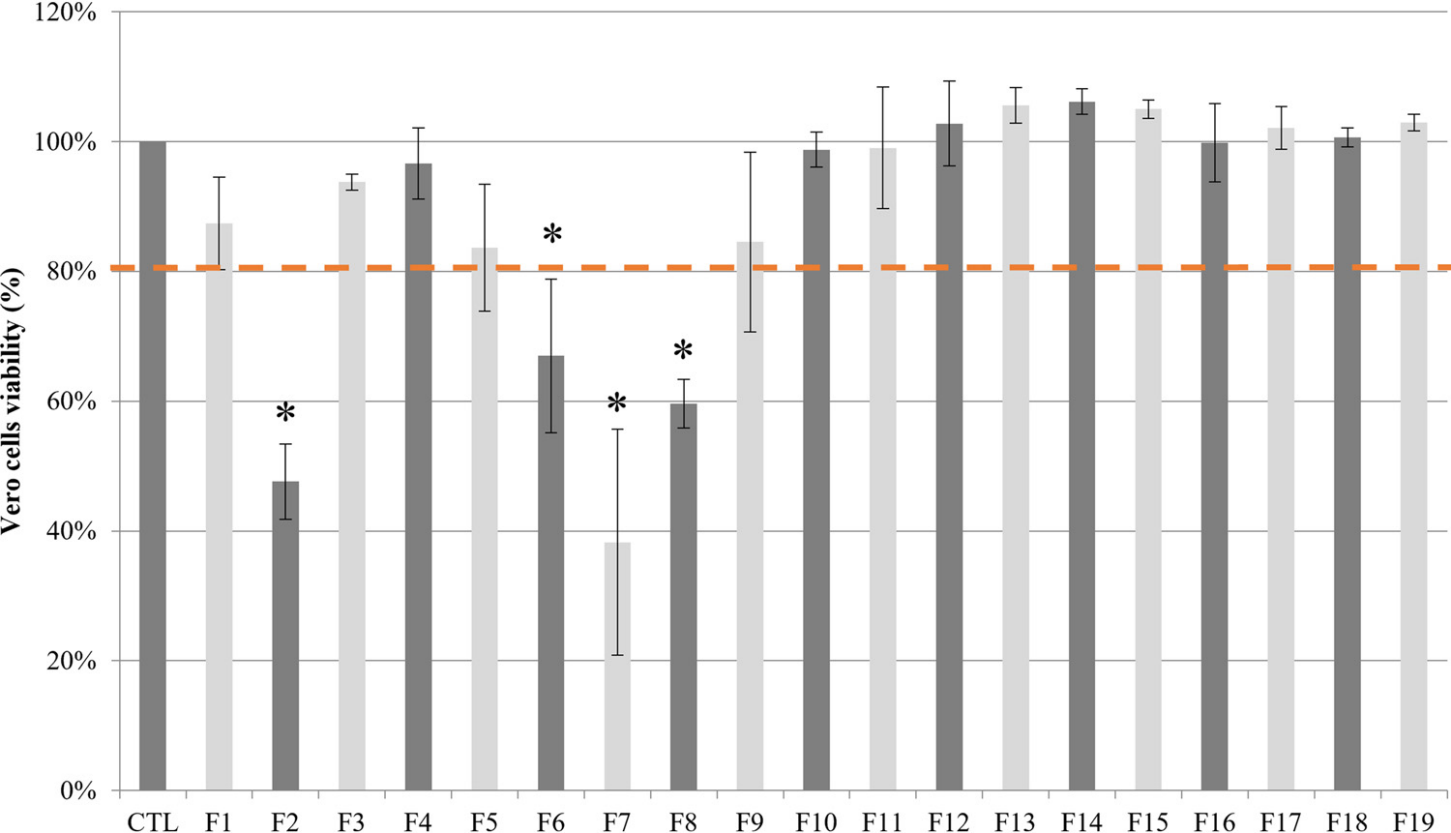
*In vitro* screening of the 19 fractions obtained from the *n*-heptane bark extract of *A. glutinosa* on Vero cells after 72 h of incubation. Each graph shows cell viability growth compared to positive control and determined by using the UptiBlue viable cell counting assay. Fractions were tested at 25 μg/mL. The dotted line indicates a 20% reduction in cell viability.

Each fraction was screened on Vero cells at 25 μg/mL to determine its cytotoxicity (Fig. 3).

Four fractions showed a significant cytotoxic effect on Vero cells (cell viability under 80%): F2 and F6-F8. Therefore, they were excluded from this study. The other fractions were tested against T. gondii.

### Screening of the 15 noncytotoxic fractions on T. gondii.

**FIG 4:**
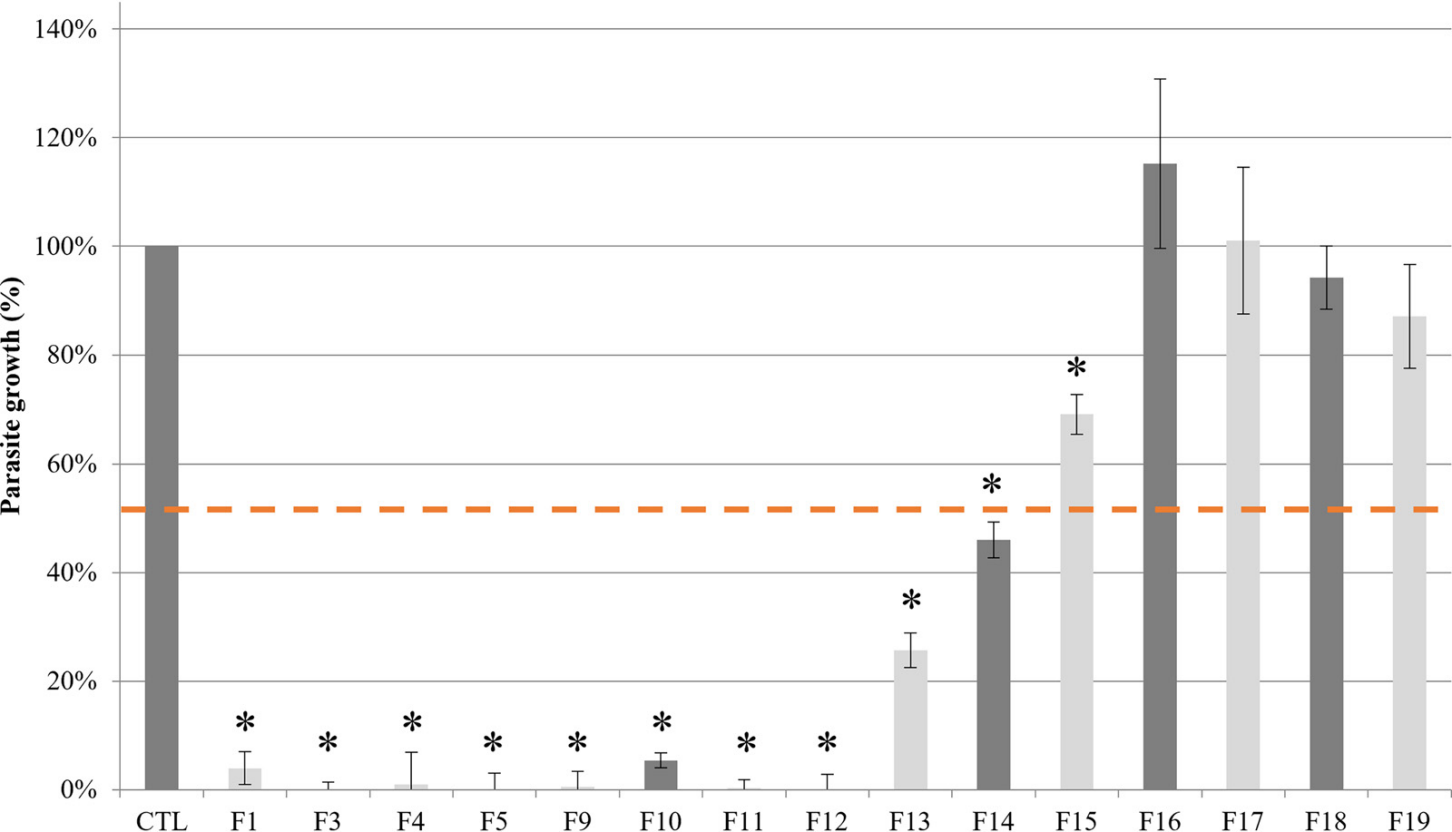
*In vitro* screening of the 15 fractions on T. gondii tachyzoites (RH strain) growth after 72 h of incubation. The tachyzoites growth was compared to a positive control and determined by enzymatic immunoassay with infected monolayers (*y* axis) versus bark extract (*x* axis). Fractions were tested at 25 μg/mL. Values are expressed as mean ± SD (*n* = 3). The dotted lines indicate a 50% reduction in parasite growth.

The 15 fractions of *A. glutinosa n*-heptane extract that showed no cytotoxicity on Vero cells were screened on T. gondii (Fig. 3). Among them, 10 fractions showed a significant anti-T. gondii effect, with more than 50% parasite growth inhibition: F1, F3, F4, F5, F9, F10, F11, F12, F13, and F14 (Fig. 4). Eight fractions were inhibiting the parasite growth under 10% (F1, F3, F4, F5, F9, F10, F11, and F12).

### CC_50_, IC_50_, and selectivity indexes determination.

The active CPC fractions on T. gondii were tested to determine their respective CC_50_, IC_50_, and selectivity indexes (Table 1). The results were confirmed microscopically. All fractions considered as active according to the previous screening were selective (SI > 4) against T. gondii. Fractions F3, F4, and F5 showed the highest selectivity indexes. Based on ^13^C NMR dereplication analysis, these fractions contained four lupane type triterpenes: betulin, betulinic acid, betulone, and betulinaldehyde.

### Chemosensitivity of T. gondii to lupane type triterpenes at 1 and 10 μM.

**FIG 5:**
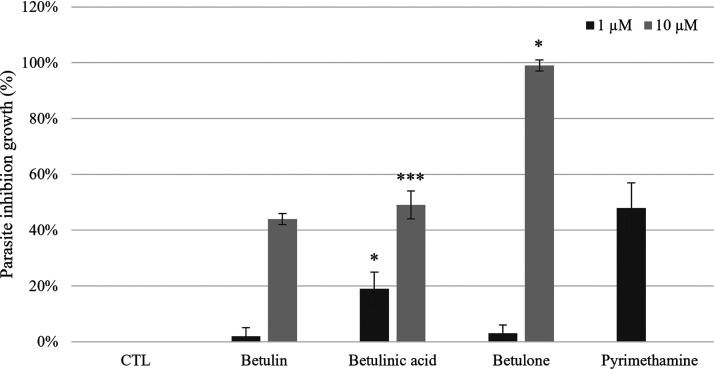
Chemosensitivity of *T.gondii* to commercially available lupane type triterpenes at 1 and 10 μM identified in *Alnus glutinosa n*-heptane extract with positive control (pyrimethamine at 1 μM) and negative control (no inhibition).

The ability of commercially available compounds (betulin, betulinic acid, betulone, and lupenone) to inhibit the growth of T. gondii at a concentration of 1 and 10 μM was evaluated. Results, shown in Fig. 5, highlighted the anti-toxoplasmic potential of betulone. Results at 1 μM did not show significant inhibition of parasite growth, while chemosensitivity at 10 μM induced an inhibition growth of 44%, 49%, and 99% for betulin, betulinic acid, and betulone, respectively. Betulone appears to be the most active triterpene. All CC_50_ associated to these compounds were above 80 μM (data not shown). Selectivity indexes were higher than for betulin, betulinic acid, betulone, and lupenone, respectively, at 10 μM.

## DISCUSSION

Barks are one of the most abundant biomasses, and their valorization is almost totally neglected. Currently, this low-value by-product is mainly burnt for combined heat and energy production. Nevertheless, potential applications could be found in many high added-value fields, including pharmaceutical and cosmetic sectors. This study aimed to investigate the potential antiparasitic activity of several extracts from 10 tree barks against T. gondii. Three solid-liquid extracts of increasing polarity (*n*-heptane, MeOH, and MeOH/H_2_O 1:1) were obtained (30 extracts), and all of them were submitted to a preliminary screening against T. gondii. The latter showed that the *n*-heptane extract of *A. glutinosa* was particularly active against T. gondii. This extract was then fractionated by CPC by using the quaternary biphasic solvent system *n*-heptane/ethyl acetate/methanol/water (9/1/9/1, vol/vol/vol/v) and chemically profiled using a ^13^C NMR-based dereplication workflow. Finally, the anti-toxoplasmic potential of the generated CPC fractions was evaluated through *in vitro* bioassays to correlate their biological activity to specialized metabolite families present in these extracts.

The fractions mainly containing oleanane or lupan triterpenes such as glutinol, betulin, and lupeol (Fractions F2, F6–F8) have proven to be cytotoxic at 25 μg/mL (< 80% cell viability) and thus were excluded from the rest of the study. Recently, a cytotoxic effect on SK-MEL cancer cell line was reported for glutinol (IC_50_ of 45.3 μM), whereas the latter was mentioned as non-cytotoxic on Vero cells (22). Cytotoxicity on Vero cells of betulin was previously reported with a CC_50_ of 160 μM (23). Finally, lupeol has only been reported as a very weak cytotoxic compound on Vero cells (24). To the best of our knowledge, no information exists concerning glutinol cytotoxicity on Vero cells. Minor compounds present in F6–F8 could also be responsible for the observed cytotoxicity, this phenomenon being today well described in the field of natural product chemistry and called residual complexity (25).

A bioassay on tachyzoites was then performed on T. gondii to determine the activity of the other noncytotoxic fractions. Fractions F1, F3–F5, and F9–F14 were active against T. gondii. Both their CC_50_ and IC_50_ were determined and then their selectivity indexes were calculated. All tested fractions were significantly selective against the parasite. The most promising activities were obtained for fractions F3–F5 and F12, with IC_50_ between 2.64 μg/mL and 3.85 μg/mL, respectively. All triterpenes found in these fractions were pentacyclic lupane type (betulin, betulinaldehyde, betulone, betulinic acid, and lupenone). Screening of commercially available compounds (betulin, betulinic acid, and betulone) at 1 μM and 10 μM was thus carried out. Lupenone, insoluble in our bioassay conditions, was not tested. As highlighted by Fig. 5, the anti-toxoplasmosis activities observed with fractions F3–F5 and F7 were confirmed on pure compounds with an inhibition activity of T. gondii growth between 44% and 99% at 10 μM. The most active compound was betulone. Little information is reported in the literature concerning the evaluation of triterpenes specifically as anti-toxoplasmosis agents. Nevertheless, a review reports the effect of pentacyclic triterpenes against tropical parasitic disease (26). One can mention for instance the action against T. gondii of maslinic acid, another pentacyclic triterpene with a carboxylic function on its C-17 position such as betulinic acid (27). The authors mention an interesting dual effect on T. gondii combing both the mobility inhibition of the parasite as well as entrance inhibition into Vero cells. The same pentacyclic triterpene was also mentioned for its parasitostatic activity toward some parasites belonging to the Apicomplexa phylum including T. gondii (28). More recently, anti-*toxoplasma* triterpenoids (29-norlupan-3,20-dione, oleanic acid acetate, and ursolic acid acetate) were isolated from *Quercus crispula* (Blume) outer bark (29). These three compounds possess also either a carboxylic substituent on C-17 or a ketone or hydroxyl on C-3, as we observed for the two most active compounds in our study: betulone and betulinic acid for which a parasite growth inhibition at 10 μM 99% and 49% were found, respectively. Endo et al. (29) also mentioned that betulin exhibited an interesting activity on *Plasmodium* with IC_50_ of 18.3 μM, but cytotoxicity against human foreskin fibroblast led to a very low selectivity (SI = 0.3). More publications report the antimalarial activity of triterpenes, *Plasmodium* sp. parasites belonging as T. gondii to the Apicomplexa phylum. For instance, *in vitro* and *in vivo* inhibition of Plasmodium falciparum growth was reported for betulinic acid—an intensively studied triterpene—and betulinic acid derivatives (30). By broadening the spectrum of the tested parasites beyond the Apicomplexa phylum, one can mention for example the lupenone, a pentacyclic triterpene with a ketone on C-3, that can induce a synergistic effect against Trypanosoma cruzi when coupled with caryophyllene oxide (31).

Considering the screening results on pure compounds and the IC_50_ measured on the most promising CPC fractions, the most interesting triterpenes possess a lupane skeleton. Oxidizing the free hydroxyl group at C-3 increases the anti-*toxoplasma* activity as clearly seen by the T. gondii inhibition growth level of betulin (or betulinaldehyde) and betulone. Moreover, the presence of a C-17 highly oxidized substituent such as a carboxylic acid group seems to counterbalance this phenomenon as highlighted by the high inhibition growth level of betulinic acid. These observations are in good agreement with the structure–activity relationship proposed by Endo et al. (29).

Finally, β-sitostenone (tetracyclic skeleton) with a ketone function in C-3, the main compound of F10-F11, showed an anti-*toxoplasma* activity of about two times lower than that observed for lupane triterpene containing fractions, but with lower cytotoxicity, suggesting a different mode of action.

In conclusion, several potent anti-*toxoplasma* triterpenes were identified in the *n*-heptane extract of *Alnus glutinosa*. Structure–activity relationships suggest that the lupane skeleton with a ketone function on C-3 or a carboxylic substituent on C-17 provides structural elements important for potent anti-*toxoplasma* activity and selectivity. Similar results obtained in Mycobacterium tuberculosis (a model that could be comparable to T. gondii since they share common characteristics (32)) support this structure–activity relationship hypothesis (33). Betulone exhibits the most interesting activity and should be a very promising candidate for the development of new anti-*toxoplasma* drugs with high efficacy and selectivity. Further synthesis of analogues or derivatives as well as *in silico* and *in vitro* investigation of their mode of action will open the door for the novel application of pentacyclic triterpenoids obtained from natural resources like temperate Northern Hemisphere tree barks.

## MATERIALS AND METHODS

### Plant materials.

France signed the Nagoya Protocol in 2011 and is a party since 2014. We received authorization from the French “Office National des Forêts” (ONF) to collect samples and use bark extracts at the University of Reims Champagne-Ardenne. Ten tree species abundantly represented in temperate forests were selected: Fagus sylvatica L., Quercus robur L., *Alnus glutinosa* (L.) Gaertn., Prunus avium (L.) L., *Acer pseudoplatanus* L., *Fraxinus excelsior* L, *Populus tremula* L, *Populus x canescens* Aiton, *Larix decidua* Mill., and *Picea abies* (L.) H.Karst. All bark samples were collected in the Champagne-Ardenne territory, in the northeast of France, in 2014. Approximately two kilograms were manually harvested from trunks 2 months after cutting over professional forestry activities. Samples were dried for 3 days at 30°C before being crushed by a hammer mill (VEM Motors GmbH, Germany) to a thin powder. Botanical identification was made according to the phenotypic characteristics of the trees, such as the shape, arrangement, and contours of leaves. Voucher specimens were deposited in the herbarium of the botanical laboratory at the faculty of Pharmacy of Reims (University of Reims Champagne-Ardenne, Reims, France): *F. sylvatica* (JH-2014-1), *Q. robur* (JH-2014-2), *A. glutinosa* (JH-2014-3), *P. avium* (JH-2014-4), *A. pseudoplatanus* (JH-2014-5), *F. excelsior* (JH-2014-6), *P. x canescens* (JH-2014-7), *L. decidua* (JH-2014-8), *P. abies* (JH-2014-9), and *P. tremula* (JH-2014-10).

### Chemicals.

Acetonitrile (CH_3_CN), methanol (MeOH), methyl-*tert*-butyl ether (M*t*BE), and *n*-heptane were purchased from Carlo Erba Reactifs SDS (Val de Reuil, France). Deuterated chloroform (chloroform-d), deuterated methanol (methanol-d_4_), lupenone (CAS: 1617-70-5), betulin (CAS: 473-98-3), and betulinic acid (CAS: 472-15-1) were purchased from Sigma-Aldrich (Saint-Quentin-Fallavier, France). Betulone (CAS: 7020-34-0) was purchased from Clickbetulin (Riga, Latvia). Deionized water was used to prepare aqueous solutions.

### Preparation of bark extracts.

Three consecutive solid-liquid extractions were performed on powdered bark with solvents of increasing polarity to cover a large chemical space. The first extraction was performed on 100 g of bark powder with 1.5 L of *n*-heptane for 18 h at room temperature under magnetic stirring. After filtration under vacuum on a sintered-glass filter of 0.4 μm, the solvent was evaporated under vacuum to give a dry *n*-heptane extract containing the less polar compounds (like fatty acids, sterols, and triterpenes). The bark residue exhausted by *n*-heptane was further dried and submitted to another solid-liquid extraction in 1.5 L of methanol for 18 h at room temperature under magnetic stirring. Methanol was evaporated under vacuum, resulting in a dry MeOH extract. The bark residue exhausted by methanol was finally submitted to a third extraction process in 1.5 L of methanol/water 1/1 (vol/vol) under the same conditions. In total, 30 extracts (three extracts for 10 trees) were obtained and were weighed after evaporation.

### Centrifugal Partition Chromatography (CPC).

The *n*-heptane extract of *A. glutinosa* bark was fractionated by CPC on a column of 303.5 mL capacity (FCPE300, Rousselet-Robatel-Kromaton, Annonay, France) containing seven partition disks engraved with 231 twin partition cells. A two-phase solvent system composed of *n-*heptane/ethyl acetate/methanol/water (9/1/9/1,) vol/vol/vol/vwas prepared in a separatory funnel. The lower phase was used as the stationary phase and pumped into the column (50 mL/min and 27 g) in the ascending mode. The rotation speed was then set at 158 g. The sample (1 g) was solubilized in 15 mL of a mixture of lower phase/upper phase in the proportions (80/20, vol/vol) and loaded into the CPC column through a 3725 Rheodyne injector valve equipped with a 15 mL sample loop. The UP was used as the mobile phase and pumped at a flow rate of 20 mL/min for 70 min. Then, the most polar compounds retained inside the column were recovered by extrusion of the stationary phase for 10 min. Fractions of 20 mL were collected over the whole experiment and combined according to their thin layer chromatography profile similarities (data not shown), resulting in a final series of 20 fractions.

### Chemical profiling of the CPC fractions.

Aliquots (up to ≈ 15 mg when possible) of CPC fractions (*n* = 20) obtained from the *n-*heptane extract of *A. glutinosa* bark were dissolved in 600 μL of CDCl_3_ and analyzed by nuclear magnetic resonance (^1^H, ^13^C, HSQC, HMBC, and COSY) at 298 K on a Bruker Avance AVIII-600 spectrometer (Karlsruhe, Germany) equipped with a TXI cryoprobe. ^13^C NMR spectra were acquired at 150.91 MHz using a standard zgpg pulse sequence with an acquisition time of 0.9 s, a relaxation delay of 3 s, and a total of 1,024 scans. After spectra processing using the TOPSPIN 3.5 software (Bruker), the absolute intensities of all ^13^C NMR signals detected in all spectra were collected by automatic peak picking. Then the ^13^C NMR spectral width (from 0 to 240 ppm) was divided into chemical shift buckets of 0.2 ppm, and the absolute intensity of the NMR peaks detected in all spectra was associated to the corresponding bucket. This step was performed using a locally developed computer script written in Python, resulting in a table with 20 columns corresponding to the CPC fractions, and 295 rows corresponding to the NMR spectral buckets for which at least one ^13^C NMR peak was detected in at least one spectrum. Hierarchical clustering analysis (HCA) was performed on the rows for data visualization of signals corresponding to major compounds contained in the *n-*heptane extract of *Alnus glutinosa* bark. The higher the intensity of ^13^C NMR peaks, the brighter the yellow color in the map. The proximity between samples was measured with the Euclidian distance, and data agglomeration was performed with Ward’s method. The resulting clusters of ^13^C NMR chemical shifts were visualized as dendrograms on a heat map (Fig. 2). The ^13^C NMR chemical shifts regrouped with the HCA were submitted to a local database containing the structures and predicted NMR chemical shifts (ACD/NMR Workbook Suite 2012 software, ACD/Labs, ON, Canada) of around 3000 natural metabolites (March 2020), to identify the corresponding chemical structures. This dereplication procedure is described in a previous publication (18). A tolerated ^13^C NMR chemical shift difference between the predicted database spectrum and the real spectrum was established at 2 ppm. Finally, each proposition given by the database was confirmed by interpretation of 1D and 2D NMR data (^1^H NMR, HSQC, HMBC, COSY).

### T. gondii strain.

RH (genotype I) strain of T. gondii was provided by the French Biological *Toxoplasma* Resource Centre (BRC *Toxoplasma*, France).

### Solubilization of extracts and fractions.

All solid-liquid extracts and CPC fractions were solubilized in DMSO. The final DMSO concentration was of 1/4000 (DMSO/medium culture, vol/vol). It has also been demonstrated that DMSO was a good lipophilic vehicle, commonly used for *in vitro* and *in vivo* experiments (34).

### Parasite growth.

T. gondii RH strain tachyzoites were cultured on Vero cell monolayers (ATCC, CCL-81) at 37°C, 5% CO_2_ in a humidified incubator. Both cells and parasites were grown in complete medium Iscove’s Modified Dulbecco’s Medium/Glutamax (IMDM) (Invitrogen, France) supplemented with 2% (vol/vol) fetal calf serum (Biowest, France) and antibiotics (100 IU/mL penicillin and 0.1 mg/mL streptomycin) (GIBCO). Host cells were infected at a 1:2 parasite to cell ratio.

### Screening of bark extracts on T. gondii.

*n-*heptane, methanol, and methanol/water 1:1 (vol/vol) bark extracts were tested on T. gondii (Fig. 1). Vero cells were seeded in a 96-wells plate, each well containing 200 μL of cell suspension with 20,000 cells. Plates were then incubated for 4 h at 37°C and 5% CO_2_. Tachyzoites grown on Vero cells were counted using a Kovas Slide counting chamber as described above. Each well was inoculated with 50 μL of parasite suspension containing 10,000 T. gondii tachyzoites (in IMDM supplemented with 2% fetal calf serum). Four wells were not inoculated and served as reference using Vero cells. These wells only contained host cells, and 50 μL of IMDM supplemented with 2% fetal calf serum were added. Four supplementary wells were inoculated with T. gondii as parasitic growth control in untreated condition and also contained 50 μL of IMDM supplemented with 2% fetal calf serum. Four last wells were inoculated under the same conditions as described above with pyrimethamine at 1 μM as control. Plates were incubated for 3 h at 37°C and 5% CO_2_. Then 25 μL of bark extract solubilized in DMSO were added to each well and tested at 100 μg/mL. Finally, plates were incubated 72 h at 37°C and 5% CO_2_ before being fixed with cold methanol.

### Cytotoxicity evaluation of CPC fractions of *n*-heptane extract of *Alnus glutinosa*.

The *in vitro* cytotoxicity of 20 CPC fractions was evaluated on Vero cell cultures using the UptiBlue viable cell-counting assay (Interchim, France) (Fig. 3). A suspension of IMDM supplemented with 2% (vol/vol) fetal calf serum containing 20,000 cells was used for each concentration. After 4 h of incubation, substances to test were deposited in wells. After 72h of incubation at 37°C and 5% CO_2_, wells were emptied and washed with cold phosphate buffered saline (Sigma-Aldrich, France). Then, 100 μL of IMDM supplemented with 2% (vol/vol) fetal calf serum and 10% (vol/vol) UptiBlue were added in each well. Afterwards, plates were incubated at 37°C for 3 h. The protocol was the same as described in the “Screening of bark extracts on T. gondii” section, except that no parasite was inoculated in wells. Spectrophotometric readings (FLUOstar Omega microplate reader, BMG Labtech, France) were made at 570 nm, corrected at 600 nm. Visual control was made as described above. A cytotoxicity threshold was arbitrarily defined at cell viability of 80%.

### Screening of active CPC fractions of *A. glutinosa* on T. gondii.

The 15 non cytotoxic CPC fractions from the *n*-heptane extract of black alder bark were tested at 25 μg/mL to avoid any cytotoxic activity on Vero cells (Fig. 4). All measures were performed in triplicate, as described in the half maximal inhibitory concentration.

### Half maximal inhibitory concentration (IC_50_).

The *in vitro* IC_50_ of the active CPC fractions against T. gondii was assessed using 96-wells plates for each fraction inhibiting at least 50% of parasite growth at 25 μg/mL within 72 h. Briefly, 200 μL aliquots of cell suspension containing 20,000 Vero cells were placed into each well and incubated at 37°C and 5% CO_2_ for 4 h to adhere. Each well, except the eight negative-control wells, was filled with 50 μL of a parasite suspension containing 10,000 T. gondii tachyzoites. The plates were incubated at 37°C and 5% CO_2_ for 3 h. Each well was filled with 25 μL of each active fraction at eight concentrations obtained by serial dilutions in the culture medium (from 25 to 0.20 μg/mL). Finally, plates were incubated at 37°C and 5% CO_2_ for 72 h before being fixed with cold methanol. Parasite growth was determined by an enzyme immunoassay (3) on the fixed infected cultures with an anti-T. gondii SAG-1-HRP conjugated monoclonal antibody (Argene Biosoft, France) and a secondary antibody coupled with horseradish peroxidase. All plates were revealed using O-Phenylenediamine Dihydrochloride (Sigma-Aldrich, France) endpoint by addition of hydrochloric acid. Spectrophotometric readings (FLUOstar Omega microplate reader, BMG Labtech, France) were made at 450 nm, corrected at 630 nm. For visual control, the last well of each condition was stained with kit RAL 555 (RAL Diagnostics, France) and examined microscopically (AxioVert 200 M, Zeiss, France) at magnification ×20 (data not shown).

### Chemosensitivity at 1 and 10 μM.

Pure purchased compounds were assessed in three wells. In a 96-wells plate, 200 μL of IMDM supplemented with 5% (vol/vol) fetal calf serum containing 20,000 Vero cells were set. After incubation for 4 h at 37°C and 5% CO_2_, 15,000 T. gondii tachyzoites were added in a volume of 50 μL in each well, except three wells for reference. After 3 h of incubation, the wells were emptied and 100 μL of drugs at a concentration of 1 μM or 10 μM in IMDM supplemented with 5% (vol/vol) fetal calf serum were added. A final incubation time of 72 h was accomplished before the determination of the parasite growth as described above (section “Half maximal inhibitory concentration (IC_50_)”).

### Selectivity indexes.

A selectivity index (SI) was calculated for each sample, as the ratio between cytotoxic and antiparasitic activities:

$$\text{S}{\text{I}}_{\text{parasite}}=\frac{\text{C}{\text{C}}_{50\;\text{Vero}}}{\text{I}{\text{C}}_{50\;\text{parasite}}}$$

The antiparasitic effect was considered selective when SI > 4.

### Statistical analysis.

Statistical analyses were performed using the R software (version 4.1.1) implemented in the RStudio IDE (version 1.4.1717). The global comparison of data associated to screening and cytotoxicity of bark extracts was performed using a Kruskal-Wallis rank sum test.

Before pairwise comparisons, data normality was assessed by a Shapiro test. Furthermore, variance homogeneity between samples were checked using a Fisher test. Pairwise comparisons of extracts or products were run out by a classical Student's *t* test when both normality and variance homogeneity null hypothesis were verified or a Welsh two samples *t* test when only the null hypothesis about data normality was verified. A Wilcoxon rank sum exact test was used for all other cases.

Significance levels for *P* values are the following: lower than 0.05 (*), lower than 0.01 (**), lower than 0.001 (***).

## References

[1] Montoya J, Liesenfeld O. 2004. Toxoplasmosis. Lancet 363:1965–1976. 10.1016/S0140-6736(04)16412-X. 15194258

[2] Luft BJ, Remington JS. 1992. Toxoplasmic encephalitis in AIDS. Clin Infect Dis 15:211–222. 10.1093/clinids/15.2.211. 1520757

[3] Doliwa C, Escotte-Binet S, Aubert D, Velard F, Schmid A, Geers R, Villena I. 2013. Induction of sulfadiazine resistance *in vitro* in *Toxoplasma gondii*. Exp Parasitol 133:131–136. 10.1016/j.exppara.2012.11.019. 23206954

[4] Montazeri M, Mehrzadi S, Sharif M, Sarvi S, Tanzifi A, Aghayan SA, Daryani A. 2018. Drug resistance in *Toxoplasma gondii*. Front Microbiol 9:2587. 10.3389/fmicb.2018.02587. 30420849 PMC6215853

[5] Reynolds MG, Oh J, Roos DS. 2001. *In vitro* generation of novel pyrimethamine resistance mutations in the *Toxoplasma gondii* dihydrofolate reductase. Antimicrob Agents Chemother 45:1271–1277. 10.1128/AAC.45.4.1271-1277.2001. 11257045 PMC90454

[6] Meneceur P, Bouldouyre M-A, Aubert D, Villena I, Menotti J, Sauvage V, Garin J-F, Derouin F. 2008. *In vitro* susceptibility of various genotypic strains of *Toxoplasma gondii* to pyrimethamine, sulfadiazine, and atovaquone. Antimicrob Agents Chemother 52:1269–1277. 10.1128/AAC.01203-07. 18212105 PMC2292506

[7] Oliveira CB, Meurer YS, Andrade JM, Costa ME, Andrade MM, Silva LA, Lanza DC, Vítor RW, Andrade-Neto VF. 2016. Pathogenicity and phenotypic sulfadiazine resistance of *Toxoplasma gondii* isolates obtained from livestock in northeastern Brazil. Mem Inst Oswaldo Cruz 111:391–398. 10.1590/0074-0276015045. 27276184 PMC4909038

[8] Newman DJ, Cragg GM. 2020. Natural products as sources of new drugs over the nearly four decades from 01/1981 to 09/2019. J Nat Prod 83:770–803. 10.1021/acs.jnatprod.9b01285. 32162523

[9] Freiburghaus F, Ogwal EN, Nkunya MH, Kaminsky R, Brun R. 1996. *In vitro* antitrypanosomal activity of African plants used in traditional medicine in Uganda to treat sleeping sickness. Trop Med Int Health 1:765–771. 10.1111/j.1365-3156.1996.tb00108.x. 8980587

[10] Bhat GP, Surolia N. 2001. *In vitro* antimalarial activity of extracts of three plants used in the traditional medicine of India. Am J Trop Med Hyg 65:304–308. 10.4269/ajtmh.2001.65.304. 11693874

[11] Nosten F, Hien TT, White NJ. 1998. Use of artemisinin derivatives for the control of malaria. Med Trop Rev Corps Sante Colon 58:45–49. 10212897

[12] Achan J, Talisuna AO, Erhart A, Yeka A, Tibenderana JK, Baliraine FN, Rosenthal PJ, D'Alessandro U. 2011. Quinine, an old anti-malarial drug in a modern world: role in the treatment of malaria. Malar J 10:144. 10.1186/1475-2875-10-144. 21609473 PMC3121651

[13] Weaver BA. 2014. How Taxol/paclitaxel kills cancer cells. Mol Biol Cell 25:2677–2681. 10.1091/mbc.E14-04-0916. 25213191 PMC4161504

[14] C Sepulveda-Arias J, A Veloza L, E Mantilla-Muriel L. 2014. Anti-*Toxoplasma* Activity of Natural Products: A Review. Recent Pat Antiinfect Drug Discov 9:186–194. 10.2174/1574891x10666150410120321. 25858302

[15] Sharif M, Sarvi S, Pagheh AS, Asfaram S, Rahimi MT, Mehrzadi S, Ahmadpour E, Gholami S, Daryani A. 2016. The efficacy of herbal medicines against *Toxoplasma gondii* during the last 3 decades: a systematic review. Can J Physiol Pharmacol 94:1237–1248. 10.1139/cjpp-2016-0039. 27564395

[16] Croteau R, Kutchan TM, Lewis NG. 2000. Natural products (secondary metabolites), p 1250–1318. *In* Buchanan BB, Gruissem W, Jones RL (ed), Biochemistry & molecular biology of plants. American Society of Plant Physiologists, Rockville, MD.

[17] Hubert J, Angelis A, Aligiannis N, Rosalia M, Abedini A, Bakiri A, Reynaud R, Nuzillard J-M, Gangloff SC, Skaltsounis A-L, Renault J-H. 2016. *In vitro* dermo-cosmetic evaluation of bark extracts from common temperate trees. Planta Med 82:1351–1358. 10.1055/s-0042-110180. 27352384

[18] Hubert J, Nuzillard J-M, Purson S, Hamzaoui M, Borie N, Reynaud R, Renault J-H. 2014. Identification of natural metabolites in mixture: a pattern recognition strategy based on (13)C NMR. Anal Chem 86:2955–2962. 10.1021/ac403223f. 24555703

[19] Berthod A, Ruiz-Angel MJ, Carda-Broch S. 2003. Elution−extrusion countercurrent chromatography. Use of the liquid nature of the stationary phase to extend the hydrophobicity window. Anal Chem 75:5886–5894. 10.1021/ac030208d. 14588030

[20] Abedini A, Chollet S, Angelis A, Borie N, Nuzillard J-M, Skaltsounis A-L, Reynaud R, Gangloff SC, Renault J-H, Hubert J. 2016. Bioactivity-guided identification of antimicrobial metabolites in *Alnus glutinosa* bark and optimization of oregonin purification by centrifugal partition chromatography. J Chromatogr B 1029–1030:121–127. 10.1016/j.jchromb.2016.07.021. 27428455

[21] Spalenka J, Hubert J, Voutquenne-Nazabadioko L, Escotte-Binet S, Borie N, Velard F, Villena I, Aubert D, Renault J-H. 2020. *In vitro* and *in vivo* activity of *Anogeissus leiocarpa* bark extract and isolated metabolites against *Toxoplasma gondii*. Planta Med 86:294–302. 10.1055/a-1088-8449. 31994148

[22] Nur-e-Alam M, Ahmed S, Yousaf M, Khan SI, Mothana RA, Al-Rehaily AJ. 2020. Isolation and characterization of cytotoxic and anti-inflammatory constituents from *Scoparia dulcis* L. J Chem Res 44:381–387. 10.1177/1747519819901100.

[23] Gong Y, Raj KM, Luscombe CA, Gadawski I, Tam T, Chu J, Gibson D, Carlson R, Sacks SL. 2004. The synergistic effects of betulin with acyclovir against herpes simplex viruses. Antiviral Res 64:127–130. 10.1016/j.antiviral.2004.05.006. 15498608

[24] Mathabe MC, Hussein AA, Nikolova RV, Basson AE, Meyer JJM, Lall N. 2008. Antibacterial activities and cytotoxicity of terpenoids isolated from *Spirostachys africana*. J Ethnopharmacol 116:194–197. 10.1016/j.jep.2007.11.017. 18191928

[25] Riihinen KR, Ou ZM, Gödecke T, Lankin DC, Pauli GF, Wu CD. 2014. The antibiofilm activity of lingonberry flavonoids against oral pathogens is a case connected to residual complexity. Fitoterapia 97:78–86. 10.1016/j.fitote.2014.05.012. 24879903

[26] Isah MB, Ibrahim MA, Mohammed A, Aliyu AB, Masola B, Coetzer THT. 2016. A systematic review of pentacyclic triterpenes and their derivatives as chemotherapeutic agents against tropical parasitic diseases. Parasitology 143:1219–1231. 10.1017/S0031182016000718. 27240847

[27] De Pablos LM, González G, Rodrigues R, García Granados A, Parra A, Osuna A. 2010. Action of a pentacyclic triterpenoid, maslinic acid, against *Toxoplasma gondii*. J Nat Prod 73:831–834. 10.1021/np900749b. 20441162

[28] Moneriz C, Marín-García P, García-Granados A, Bautista JM, Diez A, Puyet A. 2011. Parasitostatic effect of maslinic acid. I. Growth arrest of *Plasmodium falciparum* intraerythrocytic stages. Malar J 10:82. 10.1186/1475-2875-10-82. 21477369 PMC3087696

[29] Endo M, Shigetomi K, Mitsuhashi S, Igarashi M, Ubukata M. 2019. Isolation, structure determination and structure–activity relationship of anti-*toxoplasma* triterpenoids from *Quercus crispula* Blume outer bark. J Wood Sci 65:3. 10.1186/s10086-019-1782-8.

[30] de Sá MS, Costa JFO, Krettli AU, Zalis MG, Maia GL, de A, Sette IMF, Câmara C, de A, Filho JMB, Giulietti-Harley AM, Ribeiro Dos Santos R, Soares MBP. 2009. Antimalarial activity of betulinic acid and derivatives *in vitro* against *Plasmodium falciparum* and *in vivo* in *P. berghei*-infected mice. Parasitol Res 105:275–279. 10.1007/s00436-009-1394-0. 19367418

[31] Polanco-Hernández G, Escalante-Erosa F, García-Sosa K, Rosado ME, Guzmán-Marín E, Acosta-Viana KY, Giménez-Turba A, Salamanca E, Peña-Rodríguez LM. 2013. Synergistic effect of lupenone and caryophyllene oxide against *Trypanosoma cruzi*. Evid-Based Complement Altern Med 2013:435398. 10.1155/2013/435398. PMC3671683 23762135

[32] Petit-Jentreau L, Tailleux L, Coombes JL. 2017. Purinergic signaling: a common path in the macrophage response against *Mycobacterium tuberculosis* and *Toxoplasma gondii*. Front Cell Infect Microbiol 7:347. 10.3389/fcimb.2017.00347. 28824882 PMC5545599

[33] Li H, Webster D, Johnson JA, Gray CA. 2015. Anti-mycobacterial triterpenes from the Canadian medicinal plant *Alnus incana*. J Ethnopharmacol 165:148–151. 10.1016/j.jep.2015.02.042. 25725435

[34] Kelava T, Ćavar I, Čulo F. 2011. Biological actions of drug solvents. Period Biol 113(3):311–320.

