# Supplementary material for: Investigation of Antiparasitic Activity of 10 European Tree Bark Extracts on Toxoplasma gondii and Bioguided Identification of Triterpenes in Alnus glutinosa Barks
Source: Antimicrob Agents Chemother. 2022 Jan 18;66(1):e01098-21. doi: 10.1128/AAC.01098-21 (PMC8765260; doi:10.1128/AAC.01098-21)
Supplement: Supplemental file 1 — Supplemental material. Download AAC.01098-21-s0001.pdf, PDF file, 0.08 MB [file aac.01098-21-s0001.pdf]

Supplementary data

Investigation of antiparasitic activity of ten European tree bark extracts on *Toxoplasma gondii* and bioguided identification of triterpenes in *Alnus glutinosa* barks

Pierre DARME[a,b#], Jérémy SPALENKA[a*], Jane HUBERT[c], Sandie ESCOTTE-BINET[a], Laurent DEBELLE[d], Isabelle VILLENA[a,e], Charlotte SAYAGH[b], Nicolas BORIE[b], Agathe MARTINEZ[b], Benjamin BERTAUX[b], Laurence VOUTQUENNE-NAZABADIOKO[b], Jean-Hugues RENAULT[b], Dominique AUBERT[a,e]

[a] Université de Reims Champagne-Ardenne, ESCAPE EA 7510, 51097, Centre Hospitalier de Reims et Université de Reims Champagne-Ardenne, Reims, France

[b] Université de Reims Champagne-Ardenne, CNRS, ICMR 7312, 51097, Reims, France

[c] NatExplore SAS, Prouilly, France

[d] Université de Reims Champagne Ardenne, CNRS, MEDyC UMR 7369, 51097 Reims, France

[e] Centre National de Référence de la Toxoplasmose, Centre de Ressources Biologiques *Toxoplasma*, Centre Hospitalier de Reims et Université de Reims Champagne-Ardenne, Reims, France

* Current address: CEPSUP, 7bis avenue Robert Schumann, 51100, Reims FRANCE

Running Head: Anti-*toxoplasma* activity of ten European tree barks extracts

[#]Address correspondence to Pierre DARME, pierre.darme@univ-reims.fr

Illustration index

Supplementaty Table 1: Extraction yields of three extracts of ten barks 3

Supplementary Table 1. Extraction yields of three extracts of ten barks. Adapted from (1).

**Tree species**	***n*-heptane**	**MeOH**	**MeOH/H_2O 50/50 (v/v)**
F. sylvatica L. (Fagaceae)	0.3 %	2.4 %	1.8 %
Q. robur L. (Fagaceae)	0.4 %	5.6 %	3.0 %
A. glutinosa (L.) Gaertn. (Betulaceae)	1.1 %	5.6 %	1.7 %
P. avium L. (Rosaceae)	1.7 %	7.0 %	2.5 %
A. pseudoplatanus L. (Aceraceae)	1.1 %	1.4 %	1.5 %
F. excelsior L. (Oleaceae)	0.8 %	14 %	4.7 %
P. x canadensis Moench. (Salicaceae)	0.6 %	8.3 %	3.1 %
P. tremula L. (Salicaceae)	1.5 %	15.9 %	2.9 %
L. decidua Mill. (Pinaceae)	0.9 %	6.7 %	3.8 %
P. abies (L.) H. Karst. (Pinaceae)	2.5 %	18.7 %	2.8 %

References:

1. Abedini A, Chollet S, Angelis A, Borie N, Nuzillard J-M, Skaltsounis A-L, Reynaud R, Gangloff SC, Renault J-H, Hubert J. 2016. Bioactivity-guided identification of antimicrobial metabolites in Alnus glutinosa bark and optimization of oregonin purification by Centrifugal Partition Chromatography. J Chromatogr B 1029–1030:121–127.
